# The use of data mining methods for dystocia detection in Polish Holstein-Friesian Black-and-White cattle

**DOI:** 10.5713/ajas.17.0780

**Published:** 2018-04-12

**Authors:** Daniel Zaborski, Witold S. Proskura, Wilhelm Grzesiak

**Affiliations:** 1 Department of Ruminants Science, West Pomeranian University of Technology, Szczecin 71-270, Poland

**Keywords:** Dystocia, Prediction, Statistical Analysis, Dairy Cattle

## Abstract

**Objective:**

The aim of this study was to verify the usefulness of artificial neural networks (ANN), multivariate adaptive regression splines (MARS), naïve Bayes classifier (NBC), general discriminant analysis (GDA), and logistic regression (LR) for dystocia detection in Polish Holstein-Friesian Black-and-White heifers and cows and to indicate the most influential predictors of calving difficulty.

**Methods:**

A total of 1,342 and 1,699 calving records including six categorical and four continuous predictors were used. Calving category (difficult vs easy or difficult, moderate and easy) was the dependent variable.

**Results:**

The maximum sensitivity, specificity and accuracy achieved for heifers on the independent test set were 0.855 (for ANN), 0.969 (for NBC), and 0.813 (for GDA), respectively, whereas the values for cows were 0.600 (for ANN), 1.000 and 0.965 (for NBC, GDA, and LR), respectively. With the three categories of calving difficulty, the maximum overall accuracy for heifers and cows was 0.589 (for MARS) and 0.649 (for ANN), respectively. The most influential predictors for heifers were an average calving difficulty score for the dam’s sire, calving age and the mean yield of the farm, where the heifer was kept, whereas for cows, these additionally included: calf sex, the difficulty of the preceding calving, and the mean daily milk yield for the preceding lactation.

**Conclusion:**

The potential application of the investigated models in dairy cattle farming requires, however, their further improvement in order to reduce the rate of dystocia misdiagnosis and to increase detection reliability.

## INTRODUCTION

Dystocia (difficult calving) in dairy cattle, which is caused by a great number of direct and indirect factors, results in many adverse consequences [1,2]. One approach to its earlier detection is the use of statistical methods, especially those from the field of data mining. These include artificial neural networks (ANN), multivariate adaptive regression splines (MARS) and naïve Bayes classifier (NBC), among others.

The first method is based on the current knowledge of the biological nervous system and possesses some desired advantages over other classification procedures, such as: adaptability (through the learning process), robustness and insensitivity to errors [3]. A well-trained network is characterized by the ability to generalize the acquired knowledge to new cases, not used in the training process. On the other hand, MARS, which is based on generalized additive models, is built in a way similar to the recursive partitioning known from the classification and regression trees. It fits piece-wise linear basis functions to the data with each predictor range being divided into intervals using the so-called knots. The slope of the fitted basis functions between the knot pairs can vary, however, the continuity of the fitted function is retained [4]. Finally, NBC considerably simplifies the learning process by the assumption about the independence of explanatory variables given the class. Although this assumption is often violated, NBC turns out to be very effective in practice, producing comparable or even better results than other more complex models [5]. The two more traditional statistical methods used for classification are linear discriminant analysis and logistic regression (LR), utilizing continuous and categorical predictors [6,7].

So far, some data mining methods have already been used for dystocia detection in dairy cows. E.g. Zaborski and Grzesiak [8,9] applied ANN to dystocia detection in Polish Holstein-Friesian Black-and-White cattle, Zaborski et al [10] used boosted classification trees for the same purpose, Morrison et al [11,12], Basarab et al [7], Arthur et al [13], and Johnson [14] applied linear discriminant function analysis for dystocia prediction in beef heifers, whereas Piwczyński et al [15] used decision trees for analyzing factors affecting dystocia in dairy cows.

Therefore, the aim of the present study was to verify the usefulness of ANN, MARS, and NBC for dystocia detection in dairy heifers and cows and to compare them with more traditional methods, such as general discriminant analysis (GDA) and LR. The second goal was to identify the most significant predictors of calving difficulty in heifers and cows.

## MATERIALS AND METHODS

Since the present study involved only the analysis of production records routinely collected on the farms, the approval from the Local Ethics Committee on Animal Research was not necessary. A total of 1,342 and 1,699 calving records of Polish Holstein-Friesian Black-and-White heifers and cows (from the second to sixth lactation inclusive), respectively, collected between 2002 and 2013 were used. The data were derived from four commercial dairy farms located in the West Pomeranian Province. The farms were selected based on their representativeness for the relatively high-yielding dairy herds in the region. The animals were kept in free-stall barns and fed a total mixed ration. An initial set of 1,656 and 2,136 information records (for heifers and cows, respectively) was reduced after editing for missing and erroneous values and outliers. Two continuous predictors were used for heifers: AGE, calving age (in months) and MEAN, the mean calving difficulty score for the daughters of the heifer’s sire (in scores). The values of this variable were calculated from the original scores (on a five-point scale, excluding abortions). In addition, three categorical predictors were included, i.e. FARM, the category of the farm where the heifer was kept (classified according to the mean milk production as “good” equal to or above 10,000 kg milk or “poor” below 10,000 kg milk), SEASON, calving season (AW, autumn-winter from October to March and SS, spring-summer from April to September) and SEX, calf sex (M, male, F, female; twin calvings were excluded from the analysis). For cows, two additional continuous and three categorical predictors were used, i.e. CI, preceding calving interval (in days), MDM, mean daily milk yield for the previous complete lactation (in kg), PCALV2 or PCALV3, preceding calving difficulty (difficult vs easy or difficult, moderate and easy, respectively), MAST, mastitis during pregnancy (healthy vs mastitic). The mean values and standard deviations for continuous predictors are presented in Supplementary Table S1, whereas the distributions of categorical variables are given in Supplementary Table S2.

The output (dependent) variable was a calving difficulty class with two classification systems. The first one consisted of only two classes: a difficult calving (Diff) and an easy calving (Easy). The second one comprised three categories: difficult (Diff), moderate (Mod), and easy (Easy). Initially, calving difficulty was scored on a five-point scale: 1, an easy spontaneous calving, without any help from man; 2, a relatively easy calving, with help from man or mechanical equipment; 3, a complicated calving with the use of much more force than usual and/or veterinarian’s intervention; 4, a very complicated calving, including caesarean section, embryotomy and damage to the cow or calf; and 5, an abortion. Subsequently, the ordinal variable was converted to a nominal one, by assigning levels 1 and 2 to the “Easy” category and 3 and 4 to the “Diff” category in the first classification system or by assigning level 1 to the “Easy” category, 2 to the “Mod” category and 3 and 4 to the “Diff” category in the second classification system. Category 5 (abortions) was excluded from the analysis. The distribution of calving difficulty categories for heifers and cows is presented in Supplementary Table S2. The incidence of dystocia was approximately 31.8% and 3.4% in the heifer and cow datasets.

A holdout method of cross-validation was adopted in the present study. The whole dataset (1,342 and 1,699 records for heifers and cows, respectively) was randomly divided into three subsets: i) a training set (L; 50% calvings; 671 and 850 records for heifers and cows, respectively), used for model preparation, ii) a validation set (V; 25% calvings; 335 and 425 records for heifers and cows, respectively), utilized for the current monitoring of training and prevention of over-fitting, and iii) a test set (T; 25% calvings; 336 and 424 records for heifers and cows, respectively), used for the verification of the detection performance of the models. In the case of MARS, NBC, GDA and LR, the V set was combined with the L set.

The following types of ANN were investigated in the present study: linear network (LN), multilayer perceptron with one hidden layer (MLP1), multilayer perceptron with two hidden layers (MLP2) and radial basis function (RBF) network. They were trained with the following algorithms: pseudoinversion (LN), back-propagation and conjugate gradient methods (MLP1 and MLP2), k-means and k-nearest neighbor algorithms (RBF). The ANN quality was assessed with the root-mean-squared error (RMSE) on the V set. All neural models were constructed using Statistica Neural Networks software (v. 4.0F, StatSoft Inc., Tulsa, OK, USA). The best network from each category (with the lowest RMSE on the V set) was used for dystocia detection in heifers and cows on the T set.

For the MARS analysis, the following general model was used:

(0.1) $$\hat{y}=\beta_{0}+\sum_{m=1}^{M}\beta_{m}\prod_{k=1}^{K_{m}}h_{km}\left(X_{v(k,m)}\right)$$

Where, ŷ is the predicted value of the dependent variable, *β*_0_ is a constant, *h_km_*(*X_v_*_(_*_k,m_*_)_) is the basis function in which *v*(*k*,*m*) is the index of the predictor used in the *m*th component of the *k*th product, *K_m_* is the parameter limiting the order of interaction.

In the model construction, a maximum of 300 and 800 basis functions for heifers and cows, respectively, and sixth order interactions were considered. In the second stage of model building, those basis functions that did not contribute significantly to the improvement of the model performance were eliminated using the generalized cross-validation (GCV) error as a criterion:

(0.2) $$GCV(M)=\frac{1}{N}\sum_{i=1}^{N}{\left(y_{i}-\hat{y}\right)}^{2}/{\left[1-\frac{C(M)}{N}\right]}^{2}$$

Where, *N* is the number of training cases, *y_i_* is the observed value of the dependent variable, ŷ is the predicted value of the dependent variable, *C*(*M*) is the penalty function for the complexity of the model containing *M* basis functions.

The best model (with the lowest GCV error) on the training set was then used for dystocia detection on the T set.

In the NBC construction, the *a priori* probability of case membership in one of the calving difficulty classes was estimated from the training data based on Bayes’ theorem according to the following equation:

(0.3) $$P(X|y)=\prod_{j=1}^{n}P(x_{j}|y)$$

Where, X is the training vector, *y* is the value of the dependent variable (two or three classes of calving difficulty), *x_j_* is the value of the *j*th predictor conditionally independent from the remaining ones given the class, *n* is the number of independent predictors.

Probability density functions of continuous predictors were estimated from the training cases assuming their normal distribution. The new calving records were classified according to the maximum *a posteriori* probability:

(0.4) $$\hat{y}=\underset{y\in Y}{\text{arg max}}\left(P(y)\prod_{j=1}^{n}P(x_{j}|y)\right)$$

Where, ŷ is the predicted value of the dependent variable.

In the present study, GDA with the linear classification functions was also used. The number of classification functions was equal to the number of calving difficulty classes:

(0.5) $$z_{1i}=a_{10}+a_{11}x_{1i}+a_{12}x_{2i}+\ldots +a_{1n}x_{ni}$$

(0.6) $$z_{2i}=a_{20}+a_{21}x_{1i}+a_{22}x_{2i}+\ldots +a_{2n}x_{ni}$$

Where, *z*_1_*_i_* and *z*_2_*_i_* are the values of the classification functions for the ith case in the first and second class of calving difficulty, respectively (for the two-class system), *a*_10_ and *a*_20_ are the intercepts of the classification functions for the first and second class, respectively, *a*_1_*_j_* and *a*_2_*_j_* are the weights of the *j*th predictor for the first and second class, respectively.

An additional third classification function was used for the three-class system. A new case was assigned to the class, for which the value of the classification function was the highest. The assumptions of GDA were also verified and the *a priori* probability of class membership was estimated from the training data.

The last classification method used in our study was LR, whose model was given by the following formula [6]:

(0.7) $$P(Y=1|X_{1},X_{2},\ldots ,X_{n})=\frac{\text{exp}\left(a_{0}+\sum_{j=1}^{n}a_{j}X_{j}\right)}{1+\text{exp}\left(a_{0}+\sum_{j=1}^{n}a_{j}X_{j}\right)}$$

Where, *Y* is the dependent variable (calving difficulty class), *X*_1_,.., *X*_n_ are predictors, *a*_0_, *a*_j_ are regression coefficients, *n* is the number of predictors.

All statistical analyses were carried out using Statistica (v. 10, StatSoft Inc, Tulsa, OK, USA) and R (v. 3.1.0, R Core Team, R Foundation for Statistical Computing, Vienna, Austria) software. Statistical significance was declared at p<0.05.

## RESULTS

### Model quality for the two-class classification system

In the case of ANN for the heifer dataset, LN had an RMSE on the V set equal to 0.3891. The best MLP1 (RMSE = 0.3665) had a 5-2-1 structure (the number of neurons in the input, hidden and output layers, respectively), whereas the most effective MLP2 (RMSE = 0.3679) had a 5-3-6-1 structure. Finally, the structure of the best RBF network (RMSE = 0.3773) was 5-23-1. On the other hand, the final MARS model consisted of 28 basis functions and third-order interactions with the GCV error of 0.2606, while the significant effects for GDA and LR included those of AGE, MEAN, and FARM (Supplementary Tables S3, S6). The estimated parameters of the classification functions are shown in Supplementary Table S5.

For some models, the required assumptions were violated (the normal distribution of continuous predictors for NBC, and the normal distribution of residuals for GDA and LR). We found the highest sensitivity (Se; the percentage of correctly diagnosed dystocic animals) on the training set (0.8013) for MLP1 and it was significantly different from that for the non-neural classifiers (MARS, NBC, GDA, and LR). We recorded the highest specificity (Sp; the percentage of correctly diagnosed eutocic animals) (0.9927) for NBC and it differed significantly from that for all other models. We noted the highest accuracy (Acc; the percentage of correctly indicated animals from both classes) (0.8241) for MARS, but the only significant difference existed between MARS and NBC (Table 1). Finally, we observed the lowest values of Akaike information criterion (AIC) and Bayesian information criterion (BIC) (−2,034.55 and −2,009.98, respectively) for LR, and the lowest G^2^ (399.33) for MLP1 (Table 2).

For the cow dataset, LN had an RMSE equal to 0.1725. The best MLP1 (RMSE = 0.1663) and MLP2 (RMSE = 0.1646) had the 8-1-1 and 8-1-47-1 structures, respectively, whereas the structure of the most effective RBF network (RMSE = 0.1719) was 5-4-1. In the case of MARS, the final model consisted of 407 basis functions and six-order interactions with the GCV error equal to 0.0458. The estimated GDA and LR parameters are presented in Supplementary Tables S3 and S6, respectively. In both cases, the effect of MEAN, SEX, and MDM was statistically significant. The estimated parameters of the classification functions are shown in Supplementary Table S5. The assumptions that were not fulfilled included the normal distribution of continuous predictors for NBC and the normal distribution of residuals for GDA and LR. We found the highest Se on the training set of calving records (0.6512 to 0.6977) for ANN, although no statistically significant differences in Se existed among models. We observed the highest Sp (1.0000) for the non-neural classifiers, but a statistically significant difference in Sp occurred only between MLP2 and RBF. Finally, the MARS model yielded the best Acc (0.9812) and it differed significantly from that for the rest of the investigated classifiers (Table 3). We found the lowest AIC and BIC (−4,552.60 and −4,495.95, respectively) for MLP1 and the lowest G^2^ (70.24) for MARS (Table 2).

### Model quality for the three-class classification system

In the case of the three-class system for the calving records of heifers, an RMSE for LN was 0.4296. The best MLP1 (RMSE = 0.4170) had a 5-6-3 structure, whereas MLP2 with the best classification performance (RMSE = 0.4171) had a 4-15-15-3 structure. The RMSE for the most effective RBF network (a 5-9-3 structure) was 0.4260. On the other hand, the final MARS model consisted of ten basis functions and second-order interactions (GCV = 0.5236), while the effect of AGE, MEAN, and FARM was statistically significant for GDA (Supplementary Table S4). The estimated parameters of the classification functions are shown in Supplementary Table S5. However, none of the required assumptions for GDA or NBC was fulfilled. We found the highest Acc on the L+V set (0.6064) for MARS, but the differences in Acc for individual models were generally small and often non-significant (Table 4). We observed the lowest AIC and BIC values (−1,734.65 and −1,660.94, respectively) for NBC and the lowest G^2^ value (1,104.91) for MARS (Table 2).

For the cow dataset and the three-class system, LN had an RMSE on the V set equal to 0.3928. The best MLP1 (RMSE = 0.3832) and MLP2 (RMSE = 0.3834) had the 11-20-3 and 11-10-5-3 structures, respectively, whereas the structure of the best RBF network (RMSE = 0.3943) was 11-39-3. In the case of MARS, the final model consisted of 143 basis functions and five-order interactions (GCV = 0.4472), while the significant predictors for GDA included AGE, MEAN, FARM, SEX, MDM, and PCALV (Supplementary Table S4). The estimated parameters of the classification functions are shown in Supplementary Table S5. Some assumptions for GDA (the normal distribution of residuals) and NBC were not fulfilled. We found the highest Acc on the L+V set for MARS (0.7294) and it differed significantly from that for all other classifiers (Table 4). We recorded the lowest AIC and BIC values (−2,361.01 and −2,206.49, respectively) for GDA and the smallest G^2^ (870.53) for MLP1 (Table 2).

### The most influential predictors

In the two-class system for the heifer dataset, the most important predictor was MEAN followed by AGE, FARM, SEASON, and SEX (Table 5). Almost the same order of predictor importance existed in the three-class system. The only difference was that FARM was followed by AGE and that SEX was excluded from the set of predictors for MLP2 due to its error ratio below 1.0 (Table 5). For the cow dataset, the most influential predictor in the two-class system was SEX followed by MEAN, MDM, PCALV2, SEASON, AGE, MAST, CI, and FARM (Table 5). A similar order of important predictors was present for the three-class system, where MEAN was the most influential input variable followed by SEX, PCALV3, AGE (equally important), SEASON, MDM, FARM, CI, and MAST (Table 5).

### Detection performance for the two-class classification system

**Figure 1:**
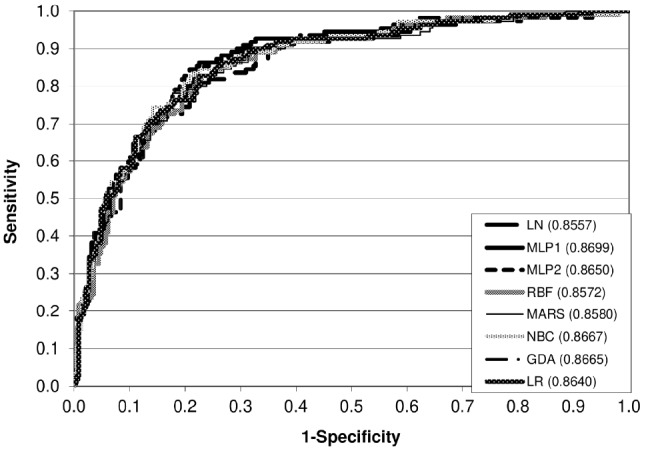
The ROC curves for different models (two-class system, heifer dataset). ROC, receiver operating characteristic; LN, linear networks; MLP1, multilayer perceptrons with one hidden layer; MLP2, multilayer perceptrons with two hidden layers; RBF, radial basis function networks; MARS, multivariate adaptive regression splines; NBC, naïve Bayes classifier; GDA, general discriminant analysis; LR, logistic regression.

We observed the highest Se on the heifer T dataset (0.8545) for MLP1 and it differed significantly from that for MARS (0.6545), NBC (0.2818), GDA (0.7000), and LR (0.6909). We found the greatest Sp (0.9690) for NBC and it was significantly different from that for all other models (Table 1). The highest Acc (0.8125) was characteristic of GDA; however, no statistically significant differences existed among different classifiers. On the other hand, NBC and MLP1 had the lowest probability of false positives (P[FP]) (0.0310) and false negatives (P[FN]) (0.1455), respectively. Finally, we found the highest *a posteriori* probability of true positives (P[PSTP]) (0.8158) for NBC and the greatest *a posteriori* probability of true negatives (P[PSTN]) (0.9162) for MLP1; however, no significant differences in the values of the former occurred. The receiver operating characteristic (ROC) curve analysis revealed that all classifiers had very similar areas under the curve (AUC) (0.86 to 0.87) (Figure 1).

**Figure 2:**
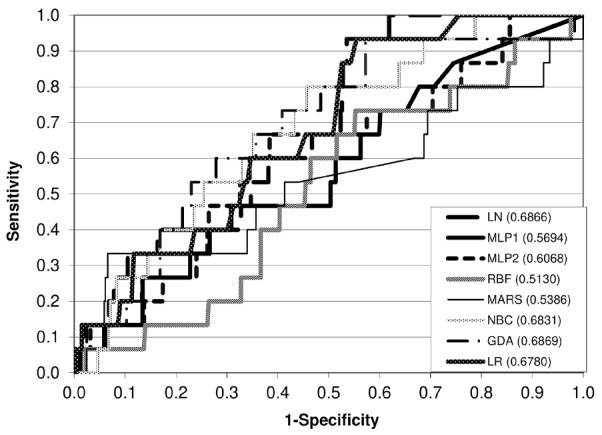
The ROC curves for different models (two-class system, cow dataset). ROC, receiver operating characteristic; LN, linear networks; MLP1, multilayer perceptrons with one hidden layer; MLP2, multilayer perceptrons with two hidden layers; RBF, radial basis function networks; MARS, multivariate adaptive regression splines; NBC, naïve Bayes classifier; GDA, general discriminant analysis; LR, logistic regression.

For the cow dataset, we found the highest Se (0.6000) for MLP2; however, no significant differences in the values of this probability existed. We recorded the greatest Sp (1.0000) for NBC, GDA, and LR (Table 3), but the only significant difference occurred between MARS and ANN. The three above-mentioned classifiers (NBC, GDA, and LR) also had the highest Acc (0.9646), which differed significantly from that for ANN, and the null P(FP). The lowest P(FN) (0.4000) and the highest P(PSTP) (0.0588) and P(PSTN) (0.9779) were all characteristic of MLP2, however, there were no significant differences in these values among different classifiers. The ROC analysis showed that the RBF network had the lowest AUC (0.51) and GDA and LN had the highest one (0.69) (Figure 2).

### Detection performance for the three-class classification system

We found the highest proportions of correctly diagnosed easy, moderate and difficult calvings in heifers obtained on the T set for GDA (0.3784), NBC (0.8000), and MARS (0.8455), respectively (Table 4). The value for GDA differed significantly from that for LN (0.2432), whereas the proportion for NBC was significantly higher that that for LN (0.4783), MLP2 (0.6696), RBF (0.5826), MARS (0.6348), and GDA (0.4522). No significant differences in the proportions of correctly indicated difficult calvings were present among models. A similar situation occurred for Acc, whose highest value was characteristic of MARS (0.5893), but, again, no significant differences in these probabilities existed.

For the cow dataset, we found the highest proportions of correctly indicated easy and moderate calvings on the T set for GDA (0.6888) and NBC (0.8779), respectively (Table 4). The value for GDA differed significantly from that for NBC (0.3622), whereas the proportion of correctly detected moderate calvings for NBC was significantly different from those for all other classifiers. For the difficult category, only MARS could correctly detect several calvings (0.0667). For all other classifiers, this proportion was null. Finally, the highest Acc on the T set was characteristic of MLP2 and RBF (0.6486), although no significant differences in this probability were present between different models.

## DISCUSSION

### Model quality

In the quality evaluation of all the models for the two-class system, Se, Sp, and Acc were used. For the three-class system, the percentage of correctly diagnosed animals from each category and the overall Acc were calculated. AIC, BIC, and G^2^ were also used for all the models. Their lower values indicated a better model.

Of the different ANN (LN, MLP1, MLP2, and RBF) used for the classification of calvings in heifers, the lowest RMSE was characteristic of MLP1 both for the two-class and three-class system, although, in the latter case, this value was almost the same as for MLP2. The MLP1 network also had the lowest G^2^ value for the two-class system indicating a good fit to the training data, although its AIC and BIC were higher than those for LR due to the greater complexity of the neural model. We observed the lowest values of these criteria in the three-class system for NBC, which indicated its superior quality in comparison with other models, although the smallest G^2^ was characteristic of MARS.

In the case of the two-class classification system, Se (i.e. the percentage of correctly diagnosed dystocic heifers) on the L and V sets obtained for all ANN types (excluding LN) in the present study (0.7918 to 0.8013) was approximately 10.0% lower than that reported by Zaborski and Grzesiak [9] in their work on dystocia detection in Polish Holstein-Friesian Black-and-White heifers. Also, Sp and Acc (0.8186 to 0.8287 and 0.8111 to 0.8201, respectively) were approximately 7.0% lower compared with the values presented in the aforementioned study, in which LN were not investigated. In a similar research on dystocia detection in dairy heifers by means of boosted classification trees [10], Se and Acc on the training set were also higher (0.894 and 0.935, respectively) in comparison with the maximum values obtained in the present work, apart from Sp, which was 5.0% lower. However, in the case of GDA, Basarab et al [7] and Arthur et al [13] reported lower Se (0.222 to 0.471 and 0.255 to 0.400, respectively), with higher Sp (0.944 to 0.980 and 0.967 to 0.980, respectively) and Acc (0.852 to 0.917 and 0.846 to 0.885, respectively) investigating dystocia detection in beef heifers using linear discriminant function analysis.

For the three-class system, the overall Acc (0.5417 to 0.6034) in the current work was relatively high. In a similar study on dystocia in Hereford heifers [14], the Acc for the five categories of calving difficulty ranged from 0.500 to 0.602 (depending on the set of predictors) and increased to 0.855 when only two categories were distinguished.

Of the different ANN types (LN, MLP1, MLP2, and RBF) used in our study for the classification of calving difficulty records in cows, the lowest RMSE was characteristic of MLP2 and MLP1 for the two-class and three-class systems, respectively, although in the latter case, the RMSE values were very similar between multilayer perceptrons. In the case of the three-class system, MLP1 also had the lowest G^2^ value indicating its good fit to the data, however, GDA had the lowest values of AIC and BIC due to its lower complexity compared with the MLP1 model. In the two-class classification system, we recorded the lowest values of AIC and BIC for MLP1 and the smallest G^2^ for MARS, which shows that MLP1 was quite effective in predicting dystocia despite its relatively high complexity.

In this system, Se on the L and V sets obtained for MLP (0.6512 to 0.6977) was lower than that (approximately 0.750) reported by Zaborski and Grzesiak [8]; however, Se for the RBF network (0.6977) in the present work was approximately 10.0% higher. Also, Sp and Acc (0.6023 to 0.6558 and 0.6055 to 0.6557, respectively) were approximately 10.0% to 15.0% lower than those in the study by Zaborski and Grzesiak [8], except for the RBF network, for which they were slightly higher. The maximum Se for all classifiers in the present study (0.6977) was approximately 18.0% lower than that reported by Zaborski et al [10], who applied boosted classification trees to dystocia detection in dairy cows, however, Sp and Acc (1.0000 and 0.9812, respectively) were almost 15.0% higher. Also, Acc obtained by Piwczyński et al [15], who used decision trees for dystocia analysis in dairy cattle, was generally lower (0.615) than that in the present work. On the other hand, Se for GDA in the current study was null, whereas it ranged from 0.157 to 0.782 or even 0.867 in similar reports [8,11,12], but Sp and Acc were higher (1.0000 and 0.9663, respectively) in comparison with the aforementioned works (0.985 and 0.917 as well as 0.812 to 0.865 and 0.824 to 0.851, respectively).

The last considered model in terms of quality measures was LR. In the study by Thirunavukkarasu and Kathiravan [6] on the prediction of conception difficulties in cows and buffaloes using LR, Se, Sp, and Acc on the training set were 0.988, 0.987, and 0.988, respectively, whereas in the work by Liu et al [16] on lameness detection in cows, the respective values for the leave-one-out cross-validation were in the ranges of 0.900 to 0.995, 0.190 to 1.000 and 0.730 to 0.996. Although, it is not possible to directly compare the above-mentioned results with those of the present study, they show, in general, the effectiveness of such models in solving various breeding issues.

In the case of the three-class system for the calving records of cows, the overall accuracy (0.6063 to 0.7294) in our study was relatively high. However, the results produced by GDA and LR for the cow dataset in the current work were clearly inferior to those obtained by means of other data mining methods (except for NBC, whose quality measures were also low) in the two-class system, whereas the values recorded for GDA in the three-class system were generally similar to those for other types of classifiers.

### The most influential predictors

Besides evaluating the detection abilities of the models on the T set, the most important predictors of calving difficulty were also identified using different criteria for different classifiers (an error ratio, the number of references to each predictor and the significance of the F and Wald statistic for ANN, MARS, GDA, and LR, respectively). Each of these methods allowed for the ordering of predictor variables according to their relative importance (an error ratio, the number of references to each predictor) or statistical significance (F and Wald statistics). The higher the error ratio and the number of references and the lower the p-values for the F and Wald statistics, the more important the predictor variable. Finally, predictor importance for all model types was presented as a rank (the lower the rank, the more important the predictor) and the five most influential predictors were further considered. However, such an analysis was not possible for NBC.

The most influential predictor for the heifer dataset was MEAN (the mean calving difficulty for the daughters of the heifer’s sire). The aim of including this variable in the models was to reflect the influence of the heifer’s sire on calving difficulty as part of the maternal effect, since the dam’s sire is partially responsible for her characteristics such as pelvic size, gestation length and body weight at calving [17]. AGE was on the second or third position for the heifer dataset, and on the second or sixth place for the cow dataset. This result is somewhat different from the sequence of input variables presented by Zaborski and Grzesiak [9], where it was ranked sixth or tenth (for heifers and cows, respectively). Also, in the work by Zaborski et al [10], it was ranked third, after gestation length and body condition score. On the other hand, Piwczyński et al [15] showed that lactation number (related to calving age) was the main factor discriminating between dystocic and eutocic animals and served as an input variable on which the first split in the classification tree was based.

In general, the greatest difference in dystocia score exists between heifers and cows [2,18], with an optimal age at first calving in dairy breeds being 22 to 24 months, which ensures optimum subsequent performance and ease of parturition [19], although a recent study on seasonally calving Holstein-Friesian heifers [20] showed that the optimum age at first calving in terms of calving difficulty was 25 to 27 months. Also, in the study by Atashi et al [18], the need for assistance, the use of considerable force and extremely difficult calvings with caesarean section, were more frequent among younger animals. Increasing age at first calving also significantly decreased the probability of calving difficulty. A significant effect of parity and calving age on the risk of dystocia or an assisted calving was confirmed by Mee et al [21], who found that the probability of an assisted calving increased linearly in heifers with a decreasing age at first calving in relation to the median age in the analyzed sample. Also, in the study by Eaglen and Bijma [22] on Dutch Holstein-Friesians, calving difficulty increased significantly with an increasing age of the dam, however, the effect of dam’s age was reduced by approximately 75.0% after the inclusion of gestation length in a statistical model, showing that it is mediated by longer gestations in older animals and may also be related to the lower fertility resulting from prolonged calving intervals. Finally, we would like to add that not all authors observed a significant effect of lactation number or calving age on dystocia incidence.

The third important predictor in the case of heifers (and the fifth or last one in the case of cows) was FARM (the mean milk production of the farm, where the animal was kept). The role of this predictor was to reflect the influence of herd-level factors on the occurrence of difficult calving in the heifer or cow. In the study by Gröhn et al [23] on the effects of host features, production and disease factors on the risk of reproductive disorders in dairy cattle, a higher herd milk yield in the current lactation was associated with an increased risk of dystocia. However, it should be mentioned that the four farms selected for the present study were characterized by relatively high milk production, which could have affected the ordering of predictor variables. Inclusion of a larger number of herds, also with lower milk production, would be warranted in the future research. The fourth (or third or fifth in the case of cows) influential input variable was calving season (SEASON). In the study on dystocia detection in dairy heifers [9], this predictor was ranked lower, whereas for cows [8], it was ranked relatively higher. On the other hand, Zaborski et al [10] found that calving season was located near the end of the sequence of significant predictors both for heifers and cows, similarly as Piwczyński et al [15]. Generally, a higher incidence of dystocia in our geographical location occurs during autumn and winter, which can be attributed to the greater extent of supervision by a farmer, an increased gestation length, calf birth weight and stillbirth rate as well as less physical exercise during this period [21]. In a recent work by Fiedlerová et al [24], a higher frequency of difficult calvings was observed in the spring (especially in April) and slightly lower during the autumn. A significant effect of calving season has also been reported by other authors [22], although it has not been proven in some studies [25].

The least influential predictor for heifers and one of the most important ones for cows was SEX (calf sex). Although it is currently possible to determine calf sex relatively early (55 to 60 dpc) using ultrasonography, this method is not routinely used for this purpose in dairy cattle farming in our country. Therefore, the inclusion of this predictor in the models can be problematic in practice. However, it was finally considered in the analysis for theoretical reasons. According to Atashi et al [18], the male sex was associated with a higher incidence of dystocia in Holstein cows and its increased risk occurred in cows giving birth to male twins. However, we did not investigate the effect of twin calvings in our study, due to the very low frequency of this calving category. On the other hand, Dhakal et al [26] found that female calves had a significantly lower probability of calving difficulty compared with male calves, but only for primiparous cows. In general, male calves have higher birth weights than female calves, which may result from longer gestations and higher androgenic hormone production [27]. A male sex is also associated with greater perinatal mortality [21]. Many other authors (e.g. Atashi et al [2]) confirmed the significant effect of calf sex on dystocia, although some investigators did not observe such a relationship.

The most important predictors for cows (apart from those already discussed for heifers) included the mean daily milk yield for the preceding lactation (MDM), and the course of previous calving (with two or three categories, depending on the classification system, PCALV). Mastitis during pregnancy (MAST) and the length of CI were the least influential predictors. In the case of MDM, it is not entirely clear whether high-yielding cows have a greater risk of dystocia [28]. Usually, an opposite association is reported in the literature, i.e. a detrimental effect of dystocia on productivity [1,2]. However, a different relationship exists for PCALV, since cows experiencing difficult parturition have a greater chance of dystocia and stillbirth at subsequent calving. For instance, Mee et al [21] found that cows with a history of dystocia at preceding calving had a 1.65 and 2.90 times higher risk of calving assistance and dystocia, respectively, during subsequent parturition. According to these authors, this result indicates that some animals are more prone to dystocia than others. A relatively high genetic correlation (0.70 to 0.80) between calving difficulty at the first and second parturition additionally supports this association. Somewhat different results were obtained by Barrier [17], who investigated two dairy herds in the UK. In the first one, the author found that the assistance (by the farmer or veterinarian) at the first calving did not result in the higher odds of such assistance at the second calving. However, assistance at the second calving was associated with a 3.4 times higher risk of assisted third calving and a 9.6 times greater chance of veterinary-assisted third parturition. In the second (smaller) herd, however, veterinary assistance at the first calving did result in a 5.3 times higher risk of assistance at the second calving, whereas assistance at the second parturition did not increase the chance of help requirement at the third calving. A similar effect was observed by Arthur et al [13], who found that Angus heifers with dystocia had significantly higher chances of its reoccurrence at the second calving.

The last two considered predictors of calving difficulty in cows were MAST and CI. MAST concerns the occurrence of mastitis during pregnancy, however, Gröhn et al [29] in their study on the influence of various diseases on culling decisions did not find any significant effect of mastitis incidence on the risk of dystocia. Although some diseases (e.g. milk fever, fatty liver syndrome) may affect the chance of calving difficulty, usually an opposite relationship is described in the literature, i.e. an increased risk of mastitis resulting from dystocia.

Finally, CI was located at the bottom of the predictor hierarchy in the studies by Zaborski and Grzesiak [8] and Zaborski et al [10]. Contrary to that finding, Fiedlerová et al [24] found a significant linear relationship between the length of CI and the occurrence of dystocia at subsequent parturition. The risk of difficult calving increased with an increasing CI but, according to the cited authors, it could be substantially reduced by the appropriate mating decisions and the avoidance of too late services. However, we would like to emphasize that there are relatively few reports on the association between preceding CI and dystocia risk, and usually an opposite relation is described, i.e. a negative effect of a difficult calving on fertility traits [1].

### Detection performance of the models

In the assessment of the detection performance of all the models for the two-class system, P(FP), P(FN), P(PSTP), and P(PSTN) were used apart from Se, Sp, and Acc. In addition, the ROC curves and AUC were determined with the higher AUC values indicating a model with greater discrimination power. For the three-class system, only the percentages of correctly classified cases from each class and the overall Acc were computed.

In the case of the two-class system for the heifer dataset, the Se on the T set for all ANN (except for LN) obtained in the present study (0.8273 to 0.8545) was higher than that (0.750 to 0.833) reported by Zaborski and Grzesiak [9], in their work on dystocia detection in dairy heifers, in which LN were not analyzed. However, Sp (0.7566 to 0.7743) and Acc (0.7798 to 0.8006) were lower than the values in the aforementioned study (0.820 to 0.876 and 0.822 to 0.861, respectively). Also, Se for all ANN (0.7818 to 0.8545) in the present work was higher than that (0.750) obtained by Zaborski et al [10], who used boosted classification trees for dystocia detection in dairy heifers, however, Se for the remaining classifiers (0.2818 to 0.7000) was lower. On the other hand, Sp for all the models (0.7566 to 0.8673), except for NBC (0.9690), as well as Acc (0.7440 to 0.8125) were lower than those (0.920 and 0.894, respectively) reported by Zaborski et al [10]. The value of P(FP) (i.e. the probability of misclassification of an eutocic animal as a dystocic one) in the present work was, in general, higher (0.0310 to 0.2434) compared with the value (0.080) obtained by Zaborski et al [10], whereas P(FN) was lower for all ANN types (0.1455 to 0.2182) and higher for the remaining classifiers (0.3000 to 0.7182). Finally, the reliability of detection made by the models in the present work (expressed as P[PSTP] and P[PSTN]) was generally higher (0.6233 to 0.8158) for the former and lower (0.7349 to 0.9162) for the latter compared with the results of Zaborski et al [10]. Moreover, the Se for GDA in the current research (0.7000) was approximately twice higher than the maximum values in the similar studies on dystocia detection in beef heifers [7,13], whereas Sp (0.8673) and Acc (0.8125) were within the ranges presented in these studies. Also, the P(FP) and P(FN) values were comparable with those reported by Basarab et al [7] and Arthur et al [13] (0.013 to 0.266 and 0.097 to 0.274 as well as 0.677 to 0.947 and 0.600 to 1.000, respectively), and the reliability of detection performed by GDA and reflected in the P(PSTP) and P(PSTN) values was relatively high in comparison with other authors (0.100 to 0.538 and 0.877 to 0.940, respectively, for Basarab et al [7] as well as 0.000 to 0.231 and 0.881 to 0.927, respectively, for Arthur et al [13]). We would like to emphasize that the most important indicator of the detection performance of the model is its Se on the T set, i.e. its ability to correctly indicate dystocic animals, and this value was quite high in our study compared with others.

The probabilities of correct detection for each of the three distinguished categories of calving difficulty and the overall Acc on the T set were acceptable (especially for the difficult class). Also, the AUC values obtained in the present study were high (approximately 0.86) and comparable with those reported by others [9,10] for dystocia detection in dairy heifers using ANN and boosted classification trees (0.85 to 0.89 and 0.81, respectively).

In the case of the two-class system for the cow dataset, the Se, Sp, and Acc on the T set for all ANN types (excluding LN) in the present study (0.4000 to 0.6000, 0.6235 to 0.6504, and 0.6156 to 0.6462, respectively) were generally lower (especially for the multilayer perceptrons) than the values reported by Zaborski and Grzesiak [8] in their work on dystocia detection in dairy cows (0.667 to 0.800, 0.611 to 0.805, and 0.615 to 0.805, respectively). Also, the maximum Se achieved on the T set for all the models (0.6000) was 15.0% lower than that in the study by Zaborski et al [10] on the use of boosted classification trees for dystocia detection in dairy cows (0.750), whereas the maximum Sp (1.0000) and Acc (0.9646) were approximately 20.0% higher. On the other hand, the values of P(FP) and P(FN) in the present work (0.0000 to 0.3765 and 0.4000 to 1.0000, respectively), were generally greater than those in the aforementioned study (0.227 and 0.250, respectively). It is especially important for the practical application of such models since a high percentage of misdiagnosed dystocic cows results in all adverse consequences associated with dystocia, both for the dam and her offspring. Also, the reliability of dystocia detection by individual models in the present work expressed as P(PSTP) was much lower (0.0375 to 0.0588) than that (0.250) recorded by Zaborski et al [10], whereas P(PSTN) was almost identical (0.9642 to 0.9779). Sensitivity for GDA in the present study was null, while the values obtained by other authors using linear discriminant function analysis [8,11,12] ranged from 0.067 to 0.571. However, Sp (1.0000) and the overall Acc (0.9646) were high and similar to the values (0.905 to 0.995 and 0.844 to 0.927, respectively) reported by others [8,11,12]. Also, P(FP) in the present work was lower (0.0000) and P(FN) was much higher (1.0000) in comparison with the rates (0.095 and 0.429, respectively) recorded by Morrison et al [12], whereas the reliability of predictions for normal calvings, i.e. P(PSTN), was slightly greater (0.9646) than that (0.905) in the above-mentioned study. However, the P(PSTP) value could not be calculated due to the absence of correctly diagnosed difficult cases. We would like to emphasize again that the most substantial measure of the model detection performance is its Se on the T set, which was unsatisfactory for cows in the current work, but one should also take into account that the frequency of difficult calvings in the cow dataset was very low (approximately 0.030) and this could have negatively affected the ability of the models to correctly diagnose such cases.

In the case of the three-class system for the calving records of cows, the percentage of correct diagnosis for the first two classes (easy and moderate) was satisfactory but it was unacceptable for the category of difficult calvings, although the overall Acc was also relatively good. The final stage of the current research was the ROC analysis. The AUC values were relatively low (0.51 to 0.69) and lower than those reported by Zaborski and Grzesiak [8] and Zaborski et al [10] for dystocia detection in dairy cows using ANN and boosted classification trees (0.63 to 0.84 and 0.86, respectively). Also, Piwczyński et al [15] in their study on dystocia and stillbirth prediction in dairy cows using decision trees obtained slightly higher values of AUC (0.61 to 0.71).

In the case of the cow dataset, the detection performance of GDA and LR on the T set in the two-class system, was, in general, much worse than that for other methods from the field of data mining (except for NBC, which was equally ineffective), whereas the probabilities for GDA in the three-class system were similar to those for other classifiers. When analyzing the performance of individual models, one should also take into account that some assumptions of their applicability were not fulfilled, which could have affected the obtained results. On the other hand, such assumptions are not required for ANN and MARS, which makes them more flexible in the task of dystocia detection.

## CONCLUSION

This study shows that different types of ANN, MARS, and NBC as well as more traditional methods such as GDA and LR are useful for the detection of difficult calvings in dairy heifers. Somewhat worse results were obtained for dystocia detection in cows, in which sensitivity was rather low, especially for the non-neural classifiers, with the acceptable levels of specificity and accuracy. This trend was confirmed in the system with three categories, in which all difficult calvings were misclassified by all the models. In general, more traditional classifiers such as GDA and LR produced results similar to more recent data mining methods, which proves their usefulness for dystocia detection. The most significant predictors for calving difficulty were an average calving difficulty score for the dam’s sire, calving age, farm category based on its mean milk production, calf sex, the difficulty of the preceding calving and the mean daily milk yield for the preceding lactation. Due to the relatively high milk production of the herds investigated in the present study, the inclusion of a larger number of herds, also with lower milk production, would be warranted in the future research. The potential application of the described methods in dairy cattle farming requires, however, their further improvement in order to eliminate the cases of misdiagnosis and increase the reliability of detection.

## Figures and Tables

**Table 1:** Probabilities for individual models on the training (n = 671), validation (n = 335), and test (n = 336) sets (two-class system, heifer dataset)

Model1)	SET2)	Se	Sp	Acc	P(FP)	P(FN)	P(PSTP)	P(PSTN)
LN	L	0.7707	0.8219	0.8063	0.1781	0.2293	0.6556	0.8907
	V	0.6875	0.8475	0.7940	0.1525	0.3125	0.6937	0.8438
	L+V	0.7413^a^	0.8302^a^	0.8022^ab^	0.1698^a^	0.2587^a^	0.6676^a^	0.8746abc
	T	0.7818^ab^	0.7832^a^	0.7827	0.2168^a^	0.2182^ab^	0.6370	0.8806^b^
MLP1	L	0.8146	0.8305	0.8256	0.1695	0.1854	0.6789	0.9106
	V	0.7768	0.8251	0.8090	0.1749	0.2232	0.6905	0.8804
	L+V	0.8013^ab^	0.8287^a^	0.8201^b^	0.1713^a^	0.1987^ab^	0.6828^a^	0.9006^a^
	T	0.8545^a^	0.7743^a^	0.8006	0.2257^a^	0.1455^a^	0.6483	0.9162^b^
MLP2	L	0.8098	0.8219	0.8182	0.1781	0.1902	0.6667	0.9076
	V	0.7589	0.8206	0.8000	0.1794	0.2411	0.6800	0.8714
	L+V	0.7918^ab^	0.8215^a^	0.8121^b^	0.1785^a^	0.2082^ab^	0.6711^a^	0.8956^ab^
	T	0.8455^a^	0.7655^a^	0.7917	0.2345^a^	0.1545^a^	0.6370	0.9105^b^
RBF	L	0.8146	0.8155	0.8152	0.1845	0.1854	0.6601	0.9091
	V	0.7589	0.8251	0.8030	0.1749	0.2411	0.6855	0.8720
	L+V	0.7950^b^	0.8186^a^	0.8111^b^	0.1814^a^	0.2050^b^	0.6684^a^	0.8967^ab^
	T	0.8273^a^	0.7566^a^	0.7798	0.2434^a^	0.1727^a^	0.6233	0.9000^b^
MARS	L+V	0.6215^c^	0.9173^b^	0.8241^b^	0.0827^b^	0.3785^c^	0.7756^a^	0.8404^bc^
	T	0.6545^b^	0.8628^b^	0.7946	0.1372^b^	0.3455^b^	0.6990	0.8369^ab^
NBC	L+V	0.2397^d^	0.9927^c^	0.7555^a^	0.0073^c^	0.7603^d^	0.9383^b^	0.7395^d^
	T	0.2818^c^	0.9690^c^	0.7440	0.0310^c^	0.7182^c^	0.8158	0.7349^a^
GDA	L+V	0.6057^c^	0.9042^b^	0.8101^b^	0.0958^b^	0.3943^c^	0.7442^a^	0.8329^c^
	T	0.7000^b^	0.8673^b^	0.8125	0.1327^b^	0.3000^b^	0.7196	0.8559^b^
LR	L+V	0.6341^c^	0.9028^b^	0.8181^b^	0.0972^b^	0.3659^c^	0.7500^a^	0.8428^bc^
	T	0.6909^b^	0.8673^b^	0.8095	0.1327^b^	0.3091^b^	0.7170	0.8522^b^

Se, sensitivity; Sp, specificity; Acc, accuracy; P(FP), false positive rate; P(FN), false negative rate; P(PSTP), *a posteriori* probability of true positives; P(PSTN), *a posteriori* probability of true negatives.

1) Model: LN, linear networks; MLP1, multilayer perceptrons with one hidden layer; MLP2, multilayer perceptrons with two hidden layers; RBF, radial basis function networks; MARS, multivariate adaptive regression splines; NBC, naïve Bayes classifier; GDA, general discriminant analysis; LR, logistic regression.

2) Dataset: L, training set; V, validation set; T, test set.

a,b,c,d Values within columns (and within sets) with different superscripts differ significantly (p<0.05).

**Table 2:** Quality measures for the models predicting calving difficulty in heifers and cows (two- and three-class system)

Model1)	Heifers			Cows		
	
	AIC	BIC	G^2^	AIC	BIC	G^2^
Two-class system						
LN	−1,887.16	−1,857.68	446.22	−4,461.26	−4,409.76	1,477.22
MLP1	−1,989.56	−1,915.85	399.33	−4,552.60	−4,495.95	1,527.00
MLP2	−1,908.76	−1,673.11	418.99	−4,257.93	−3,521.05	1,446.49
RBF	−1,701.15	−1,166.05	421.34	−4,440.15	−4,311.38	1,690.78
MARS	−1,729.76	−1,451.99	420.59	−912.21	1,108.09	70.24
NBC	−1,632.62	−1,583.48	915.50	−4,285.61	−4,192.90	-2)
GDA	−2,019.97	−1,970.83	456.65	−4,391.31	−4,298.59	-2)
LR	−2,034.55	−2,009.98	429.82	−4,409.51	−4,363.15	-2)
Three-class system						
LN	−1,663.94	−1,575.49	1,395.31	−2,308.71	−2,125.43	1,003.61
MLP1	−1,638.86	−1,365.75	1,181.25	−1,650.23	−279.29	870.53
MLP2	−621.73	750.33	1,172.18	−1,989.35	−1,064.54	892.54
RBF	−1,561.60	−1,219.08	1,282.70	−1,171.75	544.35	960.53
MARS	−1,589.96	−1,425.34	1,104.91	−1,524.42	−202.24	894.38
NBC	−1,734.65	−1,660.94	1,527.62	−2,177.10	−2,038.03	1,347.23
GDA	−1,714.63	−1,640.93	1,218.13	−2,361.01	−2,206.49	972.12

AIC, Akaike information criterion; BIC, Bayesian information criterion; G^2^, G-squared statistic.

1) Model: LN, linear networks; MLP1, multilayer perceptrons with one hidden layer; MLP2, multilayer perceptrons with two hidden layers; RBF, radial basis function networks; MARS, multivariate adaptive regression splines; NBC, naïve Bayes classifier; GDA, general discriminant analysis; LR, logistic regression.

2) Values could not be calculated.

**Table 3:** Probabilities for individual models on the training (n = 850), validation (n = 425), and test (n = 424) sets (two-class system, cow dataset)

Model1)	SET2)	Se	Sp	Acc	P(FP)	P(FN)	P(PSTP)	P(PSTN)
LN	L	0.7333	0.6439	0.6471	0.3561	0.2667	0.0701	0.9851
	V	0.6154	0.6359	0.6353	0.3641	0.3846	0.0506	0.9813
	L+V	0.6977	0.6412	0.6431^ab^	0.3588	0.3023	0.0636^a^	0.9838
	T	0.4667	0.6675^a^	0.6604^a^	0.3325^a^	0.5333	0.0490	0.9715
MLP1	L	0.7333	0.6220	0.6259	0.3780	0.2667	0.0663	0.9846
	V	0.6154	0.6578	0.6565	0.3422	0.3846	0.0537	0.9819
	L+V	0.6977	0.6339	0.6361^ab^	0.3661	0.3023	0.0624^a^	0.9836
	T	0.4667	0.6504^a^	0.6439^a^	0.3496^a^	0.5333	0.0467	0.9708
MLP2	L	0.7000	0.6634	0.6647	0.3366	0.3000	0.0707	0.9837
	V	0.5385	0.6408	0.6376	0.3592	0.4615	0.0452	0.9778
	L+V	0.6512	0.6558^a^	0.6557^a^	0.3442^a^	0.3488	0.0619^a^	0.9818
	T	0.6000	0.6479^a^	0.6462^a^	0.3521^a^	0.4000	0.0588	0.9779
RBF	L	0.7667	0.5890	0.5953	0.4110	0.2333	0.0639	0.9857
	V	0.5385	0.6286	0.6259	0.3714	0.4615	0.0438	0.9774
	L+V	0.6977	0.6023^b^	0.6055^b^	0.3977^b^	0.3023	0.0577^a^	0.9828
	T	0.4000	0.6235^a^	0.6156^a^	0.3765^a^	0.6000	0.0375	0.9659
MARS	L+V	0.4419	1.00003)	0.9812^d^	0.00003)	0.5581	1.0000^b^	0.9809
	T	0.00003)	0.9878^b^	0.9528^b^	0.0122^b^	1.00003)	0.0000	0.9642
NBC	L+V	0.00003)	1.00003)	0.9663^c^4)	0.00003)	1.00003)	-5)	0.9663
	T	0.00003)	1.00003)	0.9646^b^4)	0.00003)	1.00003)	-5)	0.9646
GDA	L+V	0.00003)	1.00003)	0.9663^c^4)	0.00003)	1.00003)	-5)	0.9663
	T	0.00003)	1.00003)	0.9646^b^4)	0.00003)	1.00003)	-5)	0.9646
LR	L+V	0.00003)	1.00003)	0.9663^c^4)	0.00003)	1.00003)	-5)	0.9663
	T	0.00003)	1.00003)	0.9646^b^4)	0.00003)	1.00003)	-5)	0.9646

Se, sensitivity; Sp, specificity; Acc, accuracy; P(FP), false positive rate; P(FN), false negative rate; P(PSTP), *a posteriori* probability of true positives; P(PSTN), *a posteriori* probability of true negatives.

1) Model: LN, linear networks; MLP1, multilayer perceptrons with one hidden layer; MLP2, multilayer perceptrons with two hidden layers; RBF, radial basis function networks; MARS, multivariate adaptive regression splines; NBC, naïve Bayes classifier; GDA, general discriminant analysis; LR, logistic regression.

2) Dataset: L, training set; V, validation set; T, test set.

3) Values of the test statistic could not be calculated.

4) Values of the test statistic could not be calculated for the comparisons between NBC, GDA, and LR.

5) P(PSTP) values could not be calculated.

a,b,c,d Values within columns (and within sets) with different superscripts differ significantly (p<0.05).

**Table 4:** Proportions of correctly classified cases for the three categories of calving difficulty in heifers and cows (three-class system)

Model1)	SET2)	Heifers				Cows			
	
		Easy	Mod	Diff	Acc	Easy	Mod	Diff	Acc
LN	L	0.2844	0.5242	0.8195	0.5365	0.6395	0.7023	0.00003)	0.6494
	V	0.2909	0.6106	0.7500	0.5522	0.6022	0.6926	0.00003)	0.6329
	L+V	0.2866^ab^	0.5512^c^	0.7950^c^	0.5417^a^	0.6275^a^	0.6990^bc^	0.00003)	0.6439^ab^
	T	0.2432^ab^	0.4783^ad^	0.8273	0.5149	0.6786^a^	0.6526^a^	0.00003)	0.6415
MLP1	L	0.2982	0.7661	0.7659	0.6140	0.6526	0.7591	0.00003)	0.6847
	V	0.3000	0.7257	0.7143	0.5821	0.6630	0.7143	0.00003)	0.6706
	L+V	0.2988^a^	0.7535^a^	0.7476abd	0.6034^b^	0.6560abc	0.7437^ab^	0.00003)	0.6800^c^
	T	0.2072abcd	0.7130^bc^	0.8000	0.5744	0.6122^a^	0.6995^a^	0.00003)	0.6344
MLP2	L	0.3303	0.6895	0.8000	0.6066	0.6526	0.7386	0.00003)	0.6741
	V	0.3182	0.6726	0.7232	0.5731	0.6575	0.7229	0.00003)	0.6729
	L+V	0.3262abc	0.6842^b^	0.7729^bc^	0.5954^b^	0.6542abc	0.7332^bc^	0.00003)	0.6737^bc^
	T	0.2613^ac^	0.6696^b^	0.8091	0.5804	0.6582^a^	0.6854^a^	0.00003)	0.6486
RBF	L	0.2936	0.7056	0.7415	0.5827	0.6658	0.7091	0.00003)	0.6647
	V	0.3182	0.6549	0.6161	0.5313	0.6077	0.6926	0.00003)	0.6353
	L+V	0.3018^ab^	0.6898^ab^	0.6972^a^	0.5656^ab^	0.6471abc	0.7034^c^	0.00003)	0.6549^bc^
	T	0.2523^ac^	0.5826abd	0.7636	0.5327	0.6633^a^	0.6808^a^	0.00003)	0.6486
MARS	L+V	0.3659^bc^	0.6648^b^	0.7886^c^	0.6064^b^	0.7094^b^	0.7854^a^	0.11633)	0.7294^d^
	T	0.2883^ac^	0.6348^ab^	0.8455	0.5893	0.6633^a^	0.6714^a^	0.06673)	0.6462
NBC	L+V	0.1677^d^	0.8283^d^	0.7066^ad^	0.5746^ab^	0.3102^d^	0.8927^d^	0.00003)	0.6063^a^
	T	0.1081^bd^	0.8000^c^	0.7636	0.5595	0.3622^b^	0.8779^b^	0.00003)	0.6085
GDA	L+V	0.4116^cd^	0.5235^c^	0.7666bcd	0.5636^ab^	0.6292^c^	0.7139^bc^	0.00003)	0.6525^bc^
	T	0.3784^cd^	0.4522^d^	0.7818	0.5357	0.6888^a^	0.6526^a^	0.00003)	0.6462

Easy, easy calving; Mod, moderate calving; Diff, difficult calving; Acc, accuracy.

1) Model: LN, linear networks; MLP1, multilayer perceptrons with one hidden layer; MLP2, multilayer perceptrons with two hidden layers; RBF, radial basis function networks; MARS, multivariate adaptive regression splines; NBC, naïve Bayes classifier; GDA, general discriminant analysis; LR, logistic regression.

2) Dataset: L, training set; V, validation set; T, test set.

3) Values of the test statistic could not be calculated.

a,b,c,d Values within columns (and within sets) with different superscripts differ significantly (p<0.05).

**Table 5:** The most influential predictors of calving difficulty for heifers and cows

Variable1)	LN	MLP1	MLP2	RBF	MARS	GDA	LR
	Heifers, two-class system						
Mean	1	1	1	1	1	1	1
Farm	2	3	3	3	4	2	2
Age	3	2	2	2	2	3	3
Season	4	4	4	4	5	4	4
Sex	5	5	5	5	3	5	5
	Heifers, three-class system						
Mean	1	1	1	1	1	1	-
Farm	2	2	2	3	2	2	-
Age	3	3	3	2	2	3	-
Season	4	4	4	5	3	4	-
Sex	5	5	Ex	4	4	5	-
	Cows, two-class system						
Mean	3	1	4	Ex	3	2	2
Farm	9	Ex	Ex	Ex	8	8	7
Age	7	5	5	Ex	1	7	8
Season	8	6	3	3	6	6	6
Sex	1	4	2	1	2	1	1
MDM	2	2	6	Ex	4	3	3
MAST	4	8	8	2	9	4	4
PCALV	5	7	1	4	7	5	5
CI	6	3	7	5	5	9	9
	Cows, three-class system						
Mean	1	1	1	3	3	1	-
Farm	5	6	6	6	6	5	-
Age	3	5	3	5	1	3	-
Season	9	3	4	1	7	8	-
Sex	2	4	5	2	5	2	-
MDM	6	7	7	7	2	4	-
MAST	8	8	9	9	8	7	-
PCALV	4	2	2	4	2	6	-
CI	7	9	8	8	4	9	-

LN, linear networks; MLP1, multilayer perceptrons with one hidden layer; MLP2, multilayer perceptrons with two hidden layers; RBF, radial basis function networks; MARS, multivariate adaptive regression splines; GDA, general discriminant analysis; LR, logistic regression.

1) Mean, mean calving difficulty for the dam’s sire; Farm, herd milk yield category; AGE, calving age; Season, calving season; Sex, calf sex; MDM, mean daily milk yield for the previous lactation; MAST, mastitis during pregnancy; PCALV, preceding calving difficulty; CI, preceding calving interval; Ex, excluded.
