# Supplementary material for: The use of data mining methods for dystocia detection in Polish Holstein-Friesian Black-and-White cattle
Source: Asian-Australas J Anim Sci. 2018 Apr 12;31(11):1700–13. doi: 10.5713/ajas.17.0780 (PMC6212759; doi:10.5713/ajas.17.0780)
Supplement: Supplementary file 1 [file ajas-31-11-1700-supplementary.pdf]

Table S1. Means and standard deviations (in parenthesis) of continuous variables within the training and test sets for heifers and cows

SET	Training (L)		Validation (V)		L+V		Test (T)		Total	
	H[1]	C[2]	H	C	H	C	H	C	H	C
n	671	850	335	425	1006	1275	336	424	1342	1699
AGE[3]	26.08	49.68	26.29	49.63	26.15	49.67	26.10	49.43	26.14	49.61
(mo)	(1.44)	(12.80)	(1.56)	(13.34)	(1.49)	(12.97)	(1.55)	(12.10)	(1.50)	(12.76)
MEAN[4]	1.81	1.75	1.79	1.77	1.80	1.76	1.85	1.72	1.82	1.75
(score)	(0.32)	(0.29)	(0.31)	(0.30)	(0.32)	(0.30)	(0.33)	(0.31)	(0.32)	(0.30)
CI[5]		408.40		405.22		407.34		406.79		407.20
(days)		(55.22)		(50.77)		(53.78)		(55.99)		(54.32)
MDM[6]		33.77		33.62		33.72		33.66		33.70
(kg)		(4.98)		(4.96)		(4.97)		(5.13)		(5.01)

[1]Heifers.

[2]Cows.

[3]Calving age.

[4]Mean calving difficulty for the dam's sire.

[5]Preceding calving interval.

[6]Mean daily milk yield for the previous lactation.

Table S2. Distributions of categorical predictors (percentages in parenthesis) within the training and test sets for heifers and cows

SET	Training (L)		Validation (V)		L+V		Test (T)		Total	
	H[1]	C[2]	H	C	H	C	H	C	H	C
n	671	850	335	425	1006	1275	336	424	1342	1699
					FARM[3]					
Good	539	698	255	357	794	1055	281	362	1075	1417
	(80.33)	(82.12)	(76.12)	(84.00)	(78.93)	(82.75)	(83.63)	(85.38)	(80.10)	(83.40)
Poor	132	152	80	68	212	220	55	62	267	282
	(19.67)	(17.88)	(23.88)	(16.00)	(21.07)	(17.25)	(16.37)	(14.62)	(19.90)	(16.60)
					SEASON[4]					
AW	359	376	169	207	528	583	154	200	682	783
	(53.50)	(44.24)	(50.45)	(48.71)	(52.49)	(45.73)	(45.83)	(47.17)	(50.82)	(46.09)
SS	312	474	166	218	478	692	182	224	660	916
	(46.50)	(55.76)	(49.55)	(51.29)	(47.51)	(54.27)	(54.17)	(52.83)	(49.18)	(53.91)
					SEX[5]					
M	310	439	166	215	476	654	159	197	635	851
	(46.20)	(51.65)	(49.55)	(50.59)	(47.32)	(51.29)	(47.32)	(46.46)	(47.32)	(50.09)
F	361	411	169	210	530	621	177	227	707	848
	(53.80)	(48.35)	(50.45)	(49.41)	(52.68)	(48.71)	(52.68)	(53.54)	(52.68)	(49.91)
					PCALV2[6]					
Easy		689		315		1004		346		1350
		(81.06)		(74.12)		(78.75)		(81.60)		(79.46)
Diff		161		110		271		78		349
		(18.94)		(25.88)		(21.25)		(18.40)		(20.54)
					PCALV3					
Easy		340		150		490		173		663
		(40.00)		(35.29)		(38.43)		(40.80)		(39.02)
Mod		349		165		514		173		687
		(41.06)		(38.82)		(40.31)		(40.80)		(40.44)

Diff	161	110	271	78	349
	(18.94)	(25.88)	(21.25)	(18.40)	(20.54)

Table 2. Cont.

MAST[7]										
Healthy		787		392		1179		399		1578
		(92.59)		(92.24)		(92.47)		(94.10)		(92.88)
Ill		63		33		96		25		121
		(7.41)		(7.76)		(7.53)		(5.90)		(7.12)
CALV2 – dependent variable (two categories)[8]										
Easy	466	820	223	412	689	1232	226	409	915	1641
	(69.45)	(96.47)	(66.57)	(96.94)	(68.49)	(96.63)	(67.26)	(96.46)	(68.18)	(96.59)
Diff	205	30	112	13	317	43	110	15	427	58
	(30.55)	(3.53)	(33.43)	(3.06)	(31.51)	(3.37)	(32.74)	(3.54)	(31.82)	(3.41)
CALV3 – dependent variable (three categories)										
Easy	218	380	110	181	328	561	111	196	439	757
	(32.49)	(44.71)	(32.84)	(42.59)	(32.60)	(44.00)	(33.04)	(46.23)	(32.71)	(44.56)
Mod	248	440	113	231	361	671	115	213	476	884
	(36.96)	(51.76)	(33.73)	(54.35)	(35.88)	(52.63)	(34.23)	(50.24)	(35.47)	(52.03)
Diff	205	30	112	13	317	43	110	15	427	58
	(30.55)	(3.53)	(33.43)	(3.06)	(31.51)	(3.37)	(32.74)	(3.54)	(31.82)	(3.41)

[1]Heifers.

[2]Cows.

[3]Herd milk yield category.

[4]Calving season (AW- autumn-winter; SS – spring-summer)

[5]Calf sex (M - male, F - female).

[6]Preceding calving difficulty (two or three categories, Mod – moderate, Diff - difficult). [7]Mastitis during pregnancy.

[8]Calving difficulty (two or three categories, Mod – moderate, Diff - difficult).

Table S3. Standardized canonical discriminant function coefficients and the significance of the effects in the general discriminant analysis (GDA) for the heifer and cow datasets (two classes)

	Coeff.[1]	Λ^2	F^3	P	Coeff.	Λ	F	P
Term[4]	Heifers				Cows			
Intercept	0.0000	0.9736	27.1567	0.0000	0.0000	0.8497	223.7238	0.0000
AGE	0.1946	0.9871	13.0551	0.0003	-0.1193	0.9997	0.3587	0.5493
MEAN	-0.8374	0.7583	318.7268	0.0000	-0.6735	0.9905	12.1119	0.0005
FARM (Good)	-0.3829	0.9491	53.6669	0.0000	-0.0887	0.9998	0.2689	0.6041
SEASON (AW)	-0.0716	0.9982	1.7613	0.1848	0.1086	0.9997	0.4334	0.5104
SEX (M)	-0.0663	0.9985	1.5519	0.2131	-0.6159	0.9889	14.2358	0.0002
CI	-	-	-	-	0.0447	0.9999	0.0653	0.7984
MDM	-	-	-	-	0.5571	0.9927	9.2597	0.0024
PCALV (Easy)	-	-	-	-	-0.3207	0.9978	2.7661	0.0965
MAST (Healthy)	-	-	-	-	-0.3079	0.9973	3.3945	0.0656

[1]Coefficient.

[2]Wilks lambda statistic.

[3]F statistic.

[4]Model term: AGE - calving age; MEAN - mean calving difficulty for the dam's sire; FARM – herd milk yield category; SEASON - calving season (AW – autumn-winter); SEX - calf sex (M – male); CI – preceding calving interval; MDM - mean daily milk yield for the previous lactation; PCALV - preceding calving difficulty (two categories), MAST - mastitis during pregnancy.

Table S4. Standardized canonical discriminant function coefficients and the significance of the effects in the general discriminant analysis (GDA) for the heifer and cow datasets (three classes)

	Coeff. 1[1]	Coeff. 2[2]	Λ[3]	F[4]	P	Coeff. 1	Coeff. 2	Λ	F	P
Term[5]			Heifers					Cows		
Intercept	0.0000	0.0000	0.9683	16.3658	0.0000	0.0000	0.0000	0.8339	125.8177	0.0000
AGE	0.1856	0.4633	0.9853	7.4600	0.0006	-0.3171	0.2998	0.9872	8.1750	0.0003
MEAN	-0.8442	0.4450	0.7506	165.9917	0.0000	0.7501	0.3506	0.9319	46.1575	0.0000
FARM (Good)	-0.3701	-0.6607	0.9461	28.4525	0.0000	-0.2075	0.2123	0.9927	4.6607	0.0096
SEASON (AW)	-0.0790	0.3782	0.9959	2.0697	0.1268	-0.0177	-0.1063	0.9997	0.1981	0.8203
SEX (M)	-0.0698	0.1743	0.9978	1.0807	0.3398	0.4044	0.4633	0.9686	20.5007	0.0000
CI	-	-	-	-	-	0.0106	-0.0540	0.9999	0.0483	0.9528
MDM	-	-	-	-	-	0.0840	-0.6873	0.9902	6.2295	0.0020
PCALV (Easy)	-	-	-	-	-	-0.0683	0.5362	0.9874	4.0221	0.0030
PCALV (Mod)	-	-	-	-	-	-0.1957	0.1065			
MAST (Healthy)	-	-	-	-	-	0.0943	0.3035	0.9965	2.1994	0.1113

[1]Coefficient 1.

[2]Coefficient 2.

[3]Wilks lambda statistic.

[4]F statistic.

[5]Model term: AGE - calving age; MEAN - mean calving difficulty for the dam's sire; FARM – herd milk yield category; SEASON - calving season (AW – autumn-winter); SEX - calf sex (M – male); CI – preceding calving interval; MDM - mean daily milk yield for the previous lactation; PCALV - preceding calving difficulty (three categories, Mod – moderate), MAST - mastitis during pregnancy.

Table S5. Estimated parameters of the classification functions in the general discriminant analysis (GDA) for the heifer and cow datasets

	Two-Class System		Three-Class System		
Term[1]	Easy	Difficult	Easy	Moderate	Difficult
			Heifers		
Intercept	-203.4774	-208.7176	-203.2263	-206.5859	-209.6515
AGE	13.3781	13.1655	13.3691	13.4425	13.1979
MEAN	32.1344	37.1757	32.1575	33.1050	37.7258
FARM (Good)	-0.7771	0.0158	-0.7868	-0.9656	-0.0950
SEASON (AW)	-3.4373	-3.3232	-3.4269	-3.3061	-3.2451
SEX (M)	-0.6946	-0.5889	-0.6904	-0.6300	-0.5509
			Cows		
Intercept	-89.0444	-93.9473	-88.5318	-91.9155	-95.0428
AGE	-0.0185	-0.0096	-0.0186	-0.0422	-0.0261
MEAN	28.2933	30.4943	28.9159	31.1736	32.7489
FARM (Good)	0.4253	0.5386	0.2345	-0.0266	0.1692
SEASON (AW)	-0.2100	-0.3152	-0.1896	-0.1971	-0.2933
SEX (M)	-0.3219	0.2757	-0.2543	0.0691	0.5516
CI	0.1706	0.1698	0.1702	0.1704	0.1696
MDM	1.5166	1.4080	1.5164	1.5417	1.4238
PCALV (Easy)	4.6270	5.0052	4.1243	3.9881	4.6078
PCALV (Mod)	-	-	1.3171	1.0781	1.1553
MAST (Healthy)	6.7200	7.2834	6.7890	6.9022	7.4355

[1]Model term: AGE - calving age; MEAN - mean calving difficulty for the dam's sire; FARM – herd milk yield category; SEASON - calving season (AW – autumn-winter); SEX - calf sex (M – male); CI – preceding calving interval; MDM - mean daily milk yield for the previous lactation; PCALV - preceding calving difficulty (two or three categories, Mod – moderate), MAST - mastitis during pregnancy.

Table S6. Estimated parameters of the logistic regression model (LR) for the heifer and cow datasets (two-class system only)

Term[1]	Estimate	Std. Error	Wald Statistic	P
		Heifers		
Intercept	-7.2142	1.8430	15.3	0.0001
AGE	-0.1877	0.0597	9.9	0.0017
MEAN	4.7057	0.3759	156.7	0.0000
FARM	3.0812	0.5937	26.9	0.0000
SEASON	-0.3028	0.1782	2.9	0.0893
SEX	-0.2212	0.1744	1.6	0.2047
		Cows		
Intercept	-5.7434	2.4916	5.3	0.0212
AGE	0.0075	0.0154	0.24	0.6243
MEAN	2.0802	0.6111	11.6	0.0007
FARM	0.2628	0.5079	0.27	0.6049
SEASON	0.2170	0.3273	0.44	0.5073
SEX	-1.3652	0.3843	12.6	0.0004
CI	-0.0003	0.0031	0.0098	0.9210
MDM	-0.1096	0.0368	8.9	0.0029
PCALV2	0.6919	0.4529	2.3	0.1266
MAST	1.6014	1.0325	2.4	0.1209

[1]Model term: AGE - calving age; MEAN - mean calving difficulty for the dam's sire; FARM – herd milk yield category; SEASON - calving season; SEX - calf sex; CI – preceding calving interval; MDM - mean daily milk yield for the previous lactation; PCALV - preceding calving difficulty (two or three categories), MAST - mastitis during pregnancy.
